# The effect of dose-interval on antibody response to mRNA COVID-19 vaccines: a prospective cohort study

**DOI:** 10.3389/fimmu.2024.1330549

**Published:** 2024-02-16

**Authors:** Nisha D. Almeida, Ian Schiller, Danbing Ke, Elsa Sakr, Maria Plesa, Sandeep Vanamala, Anne-Laure Moneger, Maria Bazan, Chiara Lucchesi, Natalia Wozniak, Jorg H. Fritz, Ciriaco A. Piccirillo, Martin Pelchat, Corey Arnold, Yannick Galipeau, Pauline S. McCluskie, Marc-Andre Langlois, Kaberi Dasgupta, Bruce D. Mazer

**Affiliations:** ^1^ Department of Medicine, Faculty of Medicine and Health Sciences, McGill University, Montreal, QC, Canada; ^2^ Health Technology Assessment Unit, McGill University Health Centre, Montreal, QC, Canada; ^3^ Centre for Outcomes Research and Evaluation, Research Institute of the McGill University Health Center, Montreal, QC, Canada; ^4^ Translational Research in Respiratory Diseases, Meakins-Christie Laboratories, Research Institute of the McGill University Health Center, Montreal, QC, Canada; ^5^ Goodman Cancer Centre, and Department of Microbiology and Immunology, McGill University, Montreal, QC, Canada; ^6^ Infectious Diseases and Immunology in Global Health Program, Research Institute of Research Institute of the McGill University Health Center, and Department of Microbiology and Immunology, McGill University, Montreal, QC, Canada; ^7^ Faculty of Medicine, Department of Biochemistry, Microbiology and Immunology and University of Ottawa, Ottawa, ON, Canada; ^8^ Department of Pediatrics, Faculty of Medicine and Health Sciences, McGill University, Montréal, QC, Canada

**Keywords:** mRNA vaccination, vaccine response, humoral immunity, COVID-19, IgG, neutralizing antibodies (NAB), vaccine schedule, adaptive immunity

## Abstract

**Background:**

Vaccination against COVID-19 is highly effective in preventing severe disease and hospitalization, but primary COVID mRNA vaccination schedules often differed from those recommended by the manufacturers due to supply chain issues. We investigated the impact of delaying the second dose on antibody responses to COVID mRNA-vaccines in a prospective cohort of health-care workers in Quebec.

**Methods:**

We recruited participants from the McGill University Health Centre who provided serum or participant-collected dried blood samples (DBS) at 28-days, 3 months, and 6 months post-second dose and at 28-days after a third dose. IgG antibodies to SARS-CoV2 spike (S), the receptor-binding domain (RBD), nucleocapsid (N) and neutralizing antibodies to the ancestral strain were assessed by enzyme-linked immunosorbent assay (ELISA). We examined associations between long (≤89 days) versus short (<89 days) between-dose intervals and antibody response through multivariable mixed-effects models adjusted for age, sex, prior covid infection status, time since vaccine dose, and assay batch.

**Findings:**

The cohort included 328 participants who received up to three vaccine doses (>80% Pfizer-BioNTech). Weighted averages of the serum (n=744) and DBS (n=216) cohort results from the multivariable models showed that IgG anti-S was 31% higher (95% CI: 12% to 53%) and IgG anti-RBD was 37% higher (95% CI: 14% to 65%) in the long *vs*. short interval participants, across all time points.

**Interpretation:**

Our study indicates that extending the covid primary series between-dose interval beyond 89 days (approximately 3 months) provides stronger antibody responses than intervals less than 89 days. Our demonstration of a more robust antibody response with a longer between dose interval is reassuring as logistical and supply challenges are navigated in low-resource settings.

## Introduction

The COVID-19 era began in December 2019, with the first reports of infection in Wuhan, China (1). Within one year, randomized controlled trial data demonstrated striking effectiveness of mRNA-based vaccines against the wild type of virus or ancestral strain, reducing rates of infection and hospitalization. Uptake of mRNA sequences coding for COVID-19 spike proteins induces vaccinated individuals to produce spike (S) proteins and form anti-S and anti-Receptor Binding Domain (RBD) antibodies. These antibodies can further be evaluated for their ability to neutralize the virus (2). Infection with SARS-CoV2 can be distinguished from the response to current mRNA vaccines by the presence of antibodies to the viral nucleocapsid (N) protein (3), induced only by infection.

Immunization schedules for vaccines frequently involve two or more injections as the primary series. In the original clinical trials of mRNA vaccines, the primary vaccine doses were spaced by 21 days for BNT162b2 from Pfizer and 28 days for mRNA-1273 from Moderna (4, 5). These relatively short intervals were likely designed to confer protection quickly, given the urgency of the pandemic. Indeed, with 21- and 28 days intervals for the BNT162b2 and mRNA-1273 vaccines respectively, there appeared to be good protection in the initial one to four months, but this was followed by a decline in vaccine efficacy against infection with waning of IgG antibodies and measures of neutralization (6). Studies of other vaccines, albeit prior to mRNA vaccine development, such as those for hepatitis B suggested that longer delays between primary series doses conferred higher antibody responses than shorter intervals (7).

In fact, for many individuals, the real-world between-dose interval was longer than 28 days because of logistical bottlenecks. Reserving vaccine doses to complete the primary series threatened to substantially delay even the partial immunity conferred by the first dose of the two-dose series for the less vulnerable, potentially contributing to persistently high levels of virus propagation. Therefore, a longer between-dose interval was adopted by jurisdictions such as the UK, Germany and Canada (8), justified by immunological studies from traditional vaccines which suggested that an initial vaccine and booster spaced too closely together might result in a suboptimal immune response (9). There were no large epidemiological studies, however, justifying or confirming this. Nonetheless, some provinces in Canada decided to rapidly vaccinate as many citizens as possible with a first dose of vaccine and to employ between-dose intervals longer than those evaluated in the original clinical trials (10).

The upper limit of this interval varied from jurisdiction to jurisdiction and was adjusted over time. In the Canadian province of Quebec, a 16-week interval between vaccines was mandated in the winter of 2021 (11). As supply improved by the summer of 2021, the interval between first and second vaccination doses was decreased to approximately eight weeks. The variations in the interval between first and second dose have allowed us to examine antibody responses over time and in response to subsequent boosters, through analysis of a cohort of health care workers and researchers in Quebec. We therefore investigated the levels of IgG antibodies to components of the mRNA vaccines administered to determine if delaying vaccines for up to 16 weeks would induce higher IgG responses compared to the shorter, 8-week interval that was ultimately recommended in Quebec when vaccine supplies improved. Due to the nature of our cohort, we were also able to include and evaluate the impact of various co-variables, including age, sex, and infection status.

## Methods

### Ethics

The Research Ethics Board of the McGill University Health Centre approved all study procedures (Protocol Numbers 2021-6747 and 2021-7534). We provided a protocol description online and invited candidates to contact study personnel through a designated email address for any questions. All participants signed an electronic (e-) informed consent form using the REDCap (Research Electronic Data Capture) secured web application.

### Design and population

This study is a prospective cohort analysis. We launched our original cohort (*Living Lab Seroprevalence Study)* in July 2020 to evaluate prevalence of symptomatic and asymptomatic COVID infection in a volunteer group of employees at the Research Institute of the McGill University Health Centre (RI-MUHC: Research Ethics Board of the MUHC; Protocol Number 2021-6747). Leveraging this infrastructure, we continued follow-up of this cohort and initiated the *Health Professional Vaccination* cohort in March 2021 (MUHC REB Protocol Numbers 2021-7534), which recruited physicians, nurses, and other clinical and research staff of the institution, with the goal of assessing immune responses to vaccination and the impact of differing between-dose intervals.

### Eligibility criteria and recruitment

Participants could enter the study prior to their first vaccine, as was the case for the original Living Lab Seroprevalence study cohort members; following their first vaccine; or no more than 7 days following their second vaccine. Those more than 7 days past their second vaccine were excluded. We promoted the study through posters, social media and word of mouth within the MUHC community.

### Questionnaires

We provided interested candidates with access to online REDCap questionnaires covering general health, exposure to COVID 19, symptoms if exposed or infected, and vaccination history, including dates and type of COVID vaccine. At each subsequent blood sampling timepoint, we forwarded participants a link to a follow-up questionnaire to collect any new information on COVID infection and vaccination.

### Blood sample collection timepoints

**Figure 1:**
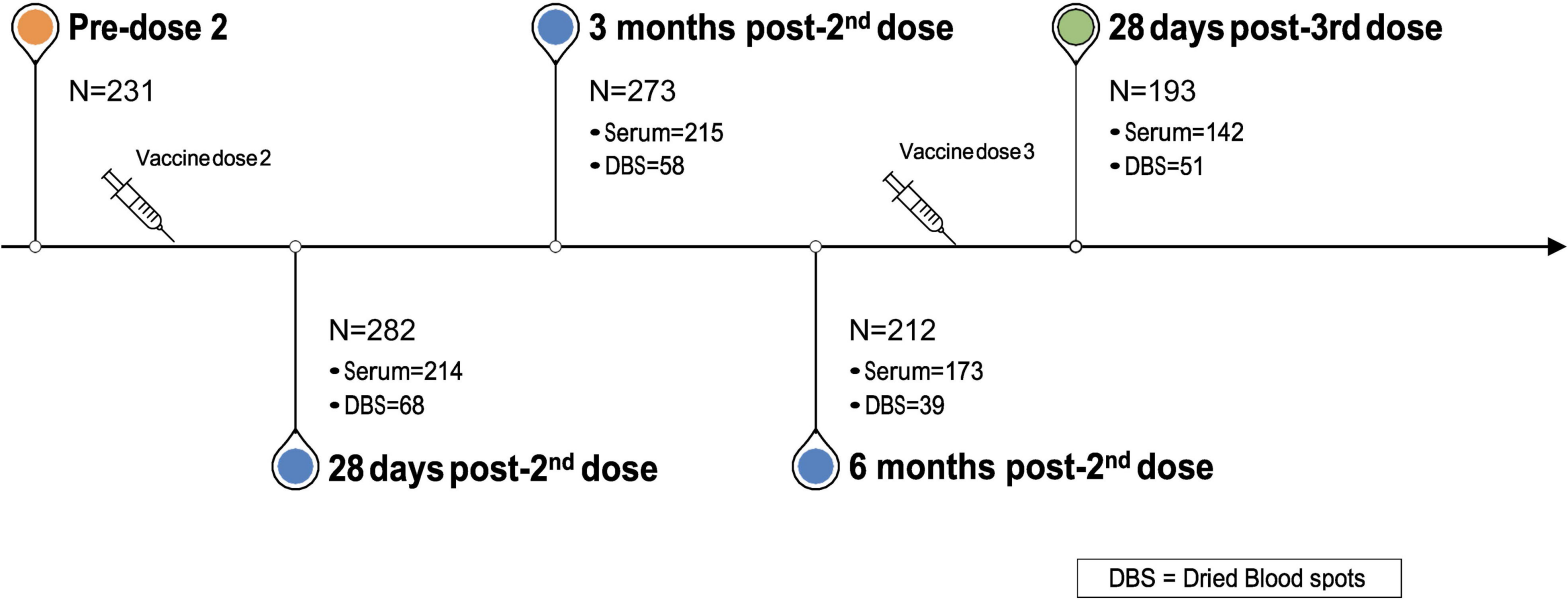
Blood (serum or DBS) samples procurement timepoints and participants’ flowchart.

Blood sample collection started in December 2020 and continues at defined intervals. In the present analysis, we evaluated antibody responses at the following time points: pre-2^nd^ vaccine dose; approximately 28 days, 3 months, and 6 months post-2^nd^ vaccine dose; and approximately 28 days post-3^rd^ vaccine dose (**Figure 1**). We analyzed data from all participants who provided at least one blood sample at any of these time points.

### Sample procurement and processing

Participants had the option to provide their samples via either venipuncture or self-collection with cards for dried blood spots (DBS), which could be performed at home and mailed to the laboratory. For those opting for venipuncture, we collected 10 ml of venous blood in clot activator red-capped blood collection tubes (BD vacutainer, Cat. #367815). We centrifuged blood samples twice at 1200 rpm for 12 minutes, collected serum from these samples and stored them in aliquots at -80°C, until processing. We provided those opting for DBS with home kits that contained ethanol swabs, lancets (Microtainer Contact-Activated Lancet, Ref. 366594, BD) and filter paper (Whatman 903 Protein Saver Cards; GE Health Care, Boston MA). We included detailed instructions asking participants to fill 5 circles on the card and mail (stamped and addressed envelope) or deliver the sample to our study center. We stored cards in desiccation boxes at room temperature until processing, as we have previously reported (12).

### Antibody measurement

We assessed IgG spike protein antibodies (S) as well as IgG antibodies specific for the receptor binding domain (RBD) and IgG nucleocapsid antibodies (N) as well as neutralizing antibodies, based upon and optimized from assays described by Colwill and colleagues (12). Automated chemiluminescent enzyme-linked immunosorbent assays (ELISA) were performed at the University of Ottawa, Faculty of Medicine, using Hamilton MicroLab Star Robotic Liquid Handlers and a 405 TS/LS LHC2 plate washer (Biotek Instruments). Incubations were performed at room temperature with shaking at 500–700 rpm. Antigens (S, RBD, aa 319–541, and N) diluted in PBS were pipetted into 384-well high-binding polystyrene Nunc plates (Thermo-Fisher Scientific, #460372;50 ng/well). We then centrifuged the plates at 216 × g for 1 minute, incubated overnight at 4°C with rocking and the wells were blocked with 80 µL of 3% w/v skim milk powder dissolved in PBST for 1 hour, followed by washing. 10 µL of samples and controls (diluted to 1% w/v skim milk powder in PBS-T) were added to each well and the plates were washed after two hours of incubation. 10 µL of diluted secondary antibodies were then added to each well. After incubation for 1 hour, we washed the wells and added 10 µL of ELISA Pico Chemiluminescent Substrate (diluted 1:2 in MilliQ H20) to each well. After a 5-minute incubation with shaking, we read the plates on a Neo2 plate reader (BioTek Instruments) at 20 ms/well with a read height of 1.0 mm, which generated luminescence values. We converted these values to WHO Binding Antibody Units (BAU), as recently reported (12). We conducted separate analyses for participants with serum and DBS blood samples as these methodologies differ, as did the units of measure (i.e., per ml for blood samples through venipuncture and per mm^2^ for DBS) (12). In addition, we employed a surrogate neutralization (sn)ELISA to measure inhibition of ACE2-spike or RBD interactions with subjects’ antibodies, as described in our recent publication (12). Results of neutralization assays are reported in International Units (IU).

### Between-dose intervals

To define long *vs*. short time intervals between the first and second vaccine doses, we examined the distribution of between-dose time intervals across the cohort. The distribution was bimodal with two clear peaks corresponding to Quebec’s change in vaccine policy from a 16-week interval to an 8-week interval (**Supplementary Figure 1**). We stratified the cohort at the mean of this distribution, labelling below the mean as ‘short interval’ and above the mean as ‘long interval,’ thus creating two groups that mirrored the vaccine experience of Quebec’s population.

### Potential confounding variables and covariates

The potential confounding variables that we selected *a priori* included age, sex, time since 2^nd^ vaccine dose (corresponding to blood sample procurement; **Figure 1**), and infection status at baseline and at subsequent visits. We defined infection status at baseline as self-reported infection and/or IgG antibody to the nucleocapsid protein at any time prior to the second dose. At each subsequent blood draw, we defined infection status by the presence of IgG anti-N at that visit or any previous visit. A further variable, sample batch, was identified in *post-hoc* analysis as a potential confounder due to considerable between-batch heterogeneity for antibody measures (**Supplementary Figure 2**). This variable represents the batching of serum samples for eventual analysis at the laboratory.

### Statistical analyses

#### Descriptive statistics

We computed descriptive statistics for the long (>89 days) and short (≤89 days) interval groups (mean and standard deviation and/or median and interquartile range for continuous variables and percentages (%) for categorical variables) for age, sex, baseline infection status (defined as infection at any point before the 2^nd^ vaccine dose), and infection status at each procurement time, separately for serum and DBS cohorts. We calculated these values with available data for each of the following timepoints: 28 days post-2^nd^ dose; 3- and 6 months post-2^nd^ dose; and 28 days post-3^rd^ dose. Most participants contributed to more than one timepoint.

At the same time points, also stratified by long and short interval, we computed both means with 95% confidence intervals (CI) and medians with interquartile range (IQR) for IgG antibodies to S and RBD, and for neutralizing antibodies. We plotted mean and median values (Sina plots) to visualize trends in antibody levels over time.

#### Multivariable model

We constructed multivariable mixed-effects models to examine associations between short *vs*. long interval and antibody responses over time, adjusted for age, sex, time since second vaccine dose, presence of infection, and inter-batch variability (lme4 package within the R statistical software [version 4.0.3) (13, 14)]. The models included repeated measurements (i.e., antibody measures over time clustered by participant) and a random intercept to allow for baseline antibody measures to vary between participants. We log-transformed antibody values to correct for skewed distributions. This model considered blood sample as the unit of analysis and included blood samples from all four timepoints. In all models, infection status was treated as a time-dependent covariate and referred to current infection or infection at a prior time point. Separate models were run for the serum and DBS cohorts, but we calculated the combined effect of the between-dose interval by taking a weighted average of the serum and DBS point estimates. Weights were inversely proportional to the variance of the point estimates.

## Results

### Participants’ characteristics

Between January and March 2021, we recruited 328 participants, who provided either serum or DBS samples at one or more timepoints (**Figure 1**): 28 days post-2^nd^ vaccine dose (n=282); 3 months post-2^nd^ dose (n=273); 6 months post-2^nd^ visit (n=212); and 28 days post-3^rd^ dose (n=193). Over 73% of participants provided serum samples at each of the four time points. 226 unique participants provided a total of 743 serum samples and 75 unique participants provided 216 DBS samples across the four time points. Mean age (SD) across the four time points ranged from 43·5 (13·2) to 46·0 (13·2) years and approximately three quarters (72·3% to 75·5%) were female (**Tables 1A-D**).

**Table 1A:** Descriptive characteristics of participants who contributed blood at 28 days post-2^nd^ dose, stratified by serum and DBS and long and short between-dose interval.

Baseline characteristics	Serum (n=214)		DBS (n=68)		All (n=282)
	Short interval (≤89 days); n=71	Long interval (>89 days); n=143	Short interval (≤89 days); n=30	Long interval (>89 days); n=38	
**Age,** mean (SD), yrs	38.8 (13.5)	45.3 (12.9)	38.5 (10.2)	49.3 (12.2)	43.5 (13.2)
**Female, %**	69.0%	74.8%	80.0%	78.9%	74.5%
Infection status at baseline*, %	7.0%	10.5%	23.3%	23.7%	12.8%
**Infection status at time of visit, %**	8.5%	11.2%	23.3%	26.3%	13.8%
**Pfizer at 1^st^ dose, %**	87.3%	97.9%	80.0%	97.4%	93.3%
**Pfizer at 2^nd^ dose, %**	90.1%	98.6%	76.7%	97.4%	94.0%
**Interval between 1^st^ and 2^nd^ vaccine dose,** mean (SD), days	63.5 (13.2)	102.4 (6.2)	58.4 (13.5)	103.3 (8.7)	88.0 (21.8)
**Interval between 2^nd^ vaccine dose and 28-day post-2^nd^ dose blood draw,** mean (SD), days	28.8 (4.4)	30.4 (3.6)	31.2 (8.0)	31.0 (6.6)	30.2 (4.9)

*Infection at any point before 2^nd^ dose.

**Table 1b:** Descriptive characteristics of participants who contributed blood at 3 months post-2nd dose, stratified by serum and DBS and long and short between-dose interval.

Baseline characteristics	Serum (n=214)		DBS (n=58)		All (n=272)
	Short interval (≤89 days); n=66	Long interval (>89 days); n=148	Short interval (≤89 days); n=26	Long interval (>89 days); n=32	
**Age, **mean (SD), yrs	38.7 (13.4)	45.7 (13.1)	38.4 (9.3)	52.2 (12.6)	44.1 (13.5)
**Female, %**	68.2%	75.0%	73.1%	75.0%	7329%
Infection status at baseline*, %	6.1%	11.5%	26.9%	25.0%	13.2%
**Infection status at time of visit, %**	7.6%	12.2%	30.8%	25.0%	14.3%
**Pfizer at 1^st^ dose, %**	86.4%	97.3%	84.6%	96.9%	93.4%
**Pfizer at 2^nd^ dose, %**	87.9%	98.6%	80.8%	87.5%	93.0%
**Interval between 1^st^ and 2^nd^ vaccine dose, **mean (SD), days	62.4 (13.7)	102.6 (7.1)	61.0 (14.1)	103.0 (7.2)	88.9 (21.6)
**Interval between 2^nd^ vaccine dose and 3-month post-2^nd^ dose blood draw, **mean (SD), days	92.5 (4.6)	90.5 (5.7)	96.0 (11.4)	94.2 (7.1)	91.9 (6.6)

*Infection at any point before 2^nd^ dose.

**Table 1C:** Descriptive characteristics of participants who contributed blood at 6 months post-2nd dose, stratified by serum and DBS and long and short between-dose interval.

Baseline characteristics	Serum (n=173)		DBS (n=39)		All (n=212)
	Short interval (≤89 days); n=33	Long interval (>89 days); n=140	Short interval (≤89 days); n=9	Long interval (>89 days); n=30	
**Age, **mean (SD), yrs	44.2 (14.0)	45.6 (13.3)	41.2 (10.4)	50.7 (11.6)	45.9 (13.2)
**Female, %**	66.7%	77.1%	77.8%	76.7%	75.5%
Infection status at baseline*, %	15.2%	12.9%	0.0%	26.7%	14.6%
**Infection status at time of visit, %**	24.2%	15.7%	22.2%	26.7%	18.9%
**Pfizer at 1^st^ dose, %**	81.8%	97.9%	88.9%	100.0%	95.3%
**Pfizer at 2^nd^ dose, %**	87.9%	98.6%	88.9%	100.0%	96.7%
**Interval between 1^st^ and 2^nd^ vaccine dose, **mean (SD), days	60.5 (16.5)	102.4 (7.1)	59.8 (11.0)	105.1 (8.2)	94.4 (19.4)
**Interval between 2^nd^ vaccine dose and 6-month post-2^nd^ dose blood draw, **mean (SD), days	187.6 (32.5)	181.8 (4.9)	182.4 (2.7)	185.2 (11.6)	183.3 (14.1)

*Infection at any point before 2^nd^ dose.

**Table 1D:** Descriptive characteristics of participants who contributed blood at 28 days post-3rd dose, stratified by serum and DBS and long and short between-dose interval.

Baseline characteristics	Serum (n=142)		DBS (n=51)		All (n=193)
	Short interval (≤89 days); n=48	Long interval (>89 days); n=94	Short interval (≤89 days); n=22	Long interval (>89 days); n=29	
**Age, **mean (SD), yrs	40.8 (14.6)	46.9 (12.3)	38.9 (10.7)	52.7 (12.8)	45.4 (13.5)
**Female, %**	64.6%	73.4%	72.7%	82.8%	72.5%
Infection status at baseline*, %	6.2%	12.8%	13.6%	27.6%	13.5%
**Infection status at time of visit, %**	31.2%	27.7%	45.5%	37.9%	32.1%
**Pfizer at 1^st^ dose, %**	81.2%	96.8%	90.9%	96.6%	92.9%
**Pfizer at 2^nd^ dose, %**	85.4%	97.9%	90.9%	96.6%	93.8%
**Interval between 1^st^ and 2^nd^ vaccine dose, **mean (SD), days	63.2 (12.5)	102.9 (7.2)	64.0 (6.8)	102.8 (8.1)	88.6 (21.0)
**Interval between 3^rd^ vaccine dose and 28-days post-3^rd^ dose blood draw, **mean (SD), days	29.8 (7.1)	–	–	–	–

*Infection at any point before 2^nd^ dose.

### Vaccination type and between-dose intervals

Over 80% of subjects received the Pfizer-BioNTech vaccine, with the remainder receiving either Moderna or AstraZeneca. The mean interval between the first and second vaccine doses was 89 days (SD: 21·2; range: 30 to 128 days) or 12·7 weeks. The distribution of the between-dose interval was bimodal, with peaks at 60 days (8·6 weeks) and 100 days (14·3 weeks). We opted to define short interval as at or below the 89 days average and long interval as above this value, such that the means of these intervals corresponded to the bimodal peaks (**Tables 1A-D**).

### COVID infection status at baseline and subsequent time points

**Figure 2:**
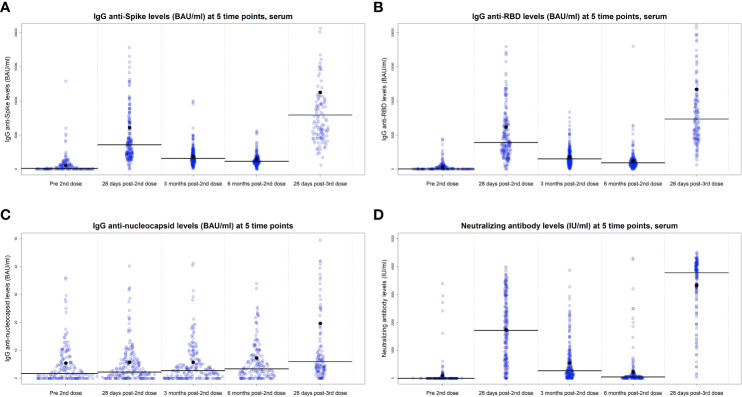
IgG antibodies levels of anti-Spike **(A)**, anti-RBD **(B)**, anti-N **(C)**, and neutralizing antibodies at 28 days post-2^nd^ dose, 3- and 6 months post-2^nd^ dose and 28 days post-3^rd^ vaccine dose, serum cohort. Lines represent the median values and dots indicate the mean. Univariate analysis, the values are BAU/ml.

Prior to their first vaccine dose, 27 participants (9·8%) were previously infected by SARS-CoV2 based on self-report and/or positive anti-N anti-body levels. The infection rate increased at each subsequent procurement time point, rising to 13·8% at 28 days after the second dose, 14·7% at 3 months post-2nd dose, 18·9% at 6 months post-2^nd^, and 31·9% 28 days post-3rd dose (**Tables 1A-D**). Thus, over the time course of the analysis, which spanned a period from January 2021 to May 2022, cumulative SARS-CoV2 infection rates rose from under 10% to over 30%. During the period of this analysis, infection status as measured by anti-N antibodies rose slowly in both groups (**Figure 2C**) and there was no statistically significant difference between the long and short vaccine dose interval groups (**Table 2**).

**Table 2:** Median antibody levels at 28 days post-2nd dose, 3 months post-2nd dose, 6 months post-2nd dose and 28-days post-3rd dose, Serum and DBS cohorts.

Median antibody levels (IQR)								
	28-day post 2^nd^ dose visit		3 months post 2^nd^ dose		6 months post 2^nd^ dose		28-day post 3^rd^ dose visit	
	Short interval (≤89 days)	Long interval (>89 days)	Short interval (≤89 days)	Long interval (>89 days)	Short interval (≤89 days)	Long interval (>89 days)	Short interval (≤89 days)	Long interval (>89 days)
**SERUM**	n=71	n=143	n=67	n=148	n=33	n=140	n=48	n=94
** IgG RBD**, BAU/ml	3458.2 (2582.4)	4733.4 (5164.5)	1607.6 (1638.8)	1419.8 (1545.4)	681.6 (1076.2)	1009.7 (805.3)	8494.7 (6650.4)	7208.5 (5768.0)
** IgG Spike**, BAU/ml	2470.9 (1933.2)	4116.2 (6060.5)	2020.2 (1599.4)	1432.2 (1265.8)	884.3 (1188.9)	1189.9 (746.6)	8870.2 (9882.5)	7746.6 (7985.1)
** IgG N**, BAU/ml	3.0 (6.9)	2.1 (6.0)	4.1 (7.0)	1.8 (4.6)	5.8 (9.1)	3.1 (7.2)	7.2 (21.7)	4.8 (13.2)
**Neutralizing** Ab, IU/ml	1713.7 (1418.5)	1718.7 (2118.5)	207.4 (541.5)	320.0 (651.7)	37.6 (167.2)	55.1 (106.3)	3694.0 (830.8)	3819.9 (996.9)
**DBS**	n=30	n=38	n=26	n=32	n=9	n=30	n=22	n=29
** IgG RBD**, BAU/ml	188.5 (48.6)	285.9 (139.1)	94.8 (75.2)	136.4 (70.1)	52.7 (29.2)	71.4 (45.0)	531.2 (506.4)	505.0 (655.7)
** IgG Spike**, BAU/ml	87.1 (41.1)	132.1 (50.9)	87.5 (64.4)	96.9 (25.6)	59.8 (20.1)	76.0 (54.0)	637.7 (1357.7)	685.3 (1619.7)
** IgG N**, BAU/ml	1.3 (1.2)	2.2 (1.0)	1.7 (0.7)	1.5 (1.1)	2.0 (0.9)	2.0 (0.9)	2.2 (2.0)	2.3 (3.4)

### Antibody response over time for the cohort

Detection of IgG antibodies against the S and RBD proteins were negligible for the limited number of participants who provided a serum sample prior to any vaccination (n=50, data not shown). The majority of the cohort, who provided a sample within ±7 days of their second vaccine dose, exhibited relatively low levels of IgG anti-S and anti-RBD prior to the second dose (n=231). As expected, antibody levels increased at 28 days post-2^nd^ dose and decreased significantly over the following 6 months (**Figures 2A, B**). For example, median IgG anti-RBD decreased from 3898 BAU/ml at 28 days post-2^nd^ dose to 1565 BAU/ml and 929 BAU/ml at 3 months and 6 months post-2^nd^ dose, respectively, and then increased to 7353 BAU/ml at 28 days post-3^rd^ dose (**Supplementary Table 1**). Similar patterns were seen for IgG anti-Spike and neutralizing antibodies, for both serum (**Figures 2B, D**) and DBS cohorts (**Supplementary Figures 3A, B**). Anti-N antibodies (**Figures 2C**; **Supplementary Figure 3C**) slowly increased over time, with highest levels in the blood procurement after the third dose, as infections increased.

### Delay of second dose influences antibody response

#### Serum cohort

There were 744 serum samples across the four time points (n= 214 at 28 days post-2nd vaccine dose; n=214 at three months post 2nd dose; n=173 at six months post-2nd dose; n=142 at 28 days post-3rd dose). Participants with the long interval between primary vaccine doses were slightly older compared to shorter interval participants (mean age 45.5 to 46.9 years *vs*. 38.6 to 44.4 years) and included a greater proportion of females (73.1% to 77.3% *vs*. 63.8% to 69.0% female) (**Tables 1A-D**).

Multivariable models indicated that participants with a long interval had IgG anti-S levels that were on average 24% higher (95% confidence interval (CI): 4% to 48%) [(1-antilog base 2(0.310))*100%] than those with the shorter between-dose intervals. They also had IgG anti-RBD levels that were 28% (95% CI: 3 to 58%) higher than those with a short interval. Although not statistically significant, serum neutralizing antibodies levels for participants with a long interval had a 35% higher point estimate (95% CI: -3 to 87%; **Table 3**).

**Table 3:** Multivariable regression analyses demonstrating the association of between-dose interval with antibody levels for participants with serum and DBS samples, separately.

	Serum (n=743)*			DBS (n=216)*		Combined effect^†^	
	**IgG RBD**	**IgG Spike**	**Neutralizing Ab**	**IgG RBD**	**IgG Spike**	**IgG RBD**	**IgG Spike**
	% change	% change	% change	% change	% change	% change	% change
	(95% CI)	(95% CI)	(95% CI)	(95% CI)	(95% CI)	(95% CI)	(95% CI)
**Interval**Long	27.7 (3.2, 58.0)	24.0 (4.1, 47.6)	34.9 (-2.7, 87.0)	66.2 (16.8, 136.5)	64.6 (16.4, 132.6)	36.8 (13.7, 64.5)	31.1 (12.0, 53.5)
Short	Reference	Reference	Reference	Reference	Reference	Reference	Reference

*Separate regression models evaluating the impact of dose interval (long vs. short) on log antibody response were run for each of the three antibodies, expressed as BAU/ml for serum and BAU/mm2 for DBS. Models were adjusted for age as a continuous variable, sex, prior infection status, batch of analysis, and timepoint. Estimates (and 95% confidence intervals shown in parentheses) represent the percent change in backtransformed antibody levels [(2β -1)*100] comparing long vs. short interval groups.

† We calculated the combined effect of the between-dose interval by taking a weighted average of the serum and DBS point estimates. Weights were inversely proportional to the variance of the point estimates.

#### DBS cohort

There were 216 DBS samples (n= 68 at 28 days post-2nd vaccine dose; n= 58 at three months post 2nd dose; n=39 at six months post-2nd dose; n=51 at 28 days post-3rd dose). Participants with a long interval between the primary vaccine doses who provided DBS samples were slightly older than short interval DBS sample participants but were generally similar in the proportion of females (75.0% to 82.8% *vs*. 72.7% to 78.9%; **Tables 1A-D**).

Similar to the serum cohort, multivariable analyses demonstrated higher antibody levels in the long *vs*. short interval participants for both IgG anti-S (65% higher, 95% CI: 16% to 133%) and IgG anti-RBD levels (66% higher, 95% CI: 17% to 137%; **Table 3**).

#### Combined effect

The weighted average of the multivariable estimates from the combined DBS and serum cohorts showed that IgG anti-S was 31% higher (95% CI: 12% to 53%) and IgG anti-RBD was 37% higher (95% CI: 14% to 65%) in the long *vs*. short interval participants.

#### Association between other covariates and antibody response

Sex, infection status and age: Our fully adjusted models of participants who contributed serum samples indicate that mean IgG anti-Spike responses were 25% higher (95% CI: 6 to 48%); IgG anti-RBD were 37% higher (95% CI: 11 to 68%); and neutralizing antibody levels were 52% higher (95% CI: 11 to 108%) in females compared to males (**Supplementary Table 2**). In the smaller subset within the DBS cohort, no significant differences in antibody responses between males and females were detected.

As expected, having prior SARS-CoV2 infection resulted in higher antibody responses for IgG anti-S, IgG anti-RBD, and neutralizing antibodies, for both the serum and DBS cohorts. For those with prior SARS-CoV2 infection, serum IgG anti-S levels were 53% higher (95% CI: 29 to 82%), IgG anti-RBD were 66% higher (95% CI: 34 to 106%), and neutralizing antibodies were 87% higher; (95% CI: 35 to 159%), than those without prior infection.

IgG anti-S, IgG anti-RBD, and neutralizing antibody levels also were age dependent; the levels decreased with increasing age, for both the serum and DBS cohorts (**Supplementary Table 2**). Every 10-year increase in age was associated with a 16% (95% CI: 12 to 14%) decrease in serum IgG anti-RBD levels [(1-antilog base 2(-0.025*10))*100%], and a 10% (95% CI: 5 to 15%) decrease in serum IgG anti-S levels.

Inter-batch variability: The final adjusted model indicates that differences in antibody measures between batches was not fully explained by the other variables in the model. This heterogeneity occurred in measurement of serum but not DBS samples.

## Discussion

Our large prospective cohort study demonstrates that subjects with 89 days or more between the two primary series doses of the SARS-CoV2 mRNA vaccine had higher levels of anti-S and anti-RBD IgG binding antibodies than those with less than 89 days between doses, after accounting for factors such as age, sex, and infection status. This difference was seen whether the subject provided a conventional serum sample, or a DBS sample. It was evident across time points that we evaluated post 2^nd^ dose, including the response following the third booster dose. The long-interval group received their 2^nd^ dose an average of approximately 100 days following the first dose, compared to 60 days between doses for the short-interval group.

Smaller studies evaluating vaccine delay have been carried out in the UK, Germany and in other Canadian provinces such as British Columbia and Ontario (8, 15–19). Delays in vaccine administration have ranged from over 28 days to up to 112 days. Studies indicate that intervals greater than 21-28 days lead to higher peak IgG anti-S and anti-RBD binding antibodies, in addition to other observations including improved neutralizing capacity and antibody dependent cellular cytotoxicity (20). However, in addition to being small and unadjusted for potential confounding factors, most studies only investigated the effects of vaccine administration interval on the primary series of vaccination; our study analyzed the impact up to and including 28-days following the third, booster dose.

Recently, data from a smaller cohort of just over 100 subjects (45 in the long interval group) suggested that while an interval of over 100 days elicits better humoral responses than 21 days, the effect of the longer interval was lost when evaluating the third booster dose of vaccine (21). This was not the case in our samples. Differences in sample size, as well as analysis parameters to address covariates may account for the difference in outcomes following the third vaccine dose compared to our cohort.

Due to limitations on travel and access for some members of the cohort, we gave subjects the opportunity to participate remotely by providing Whatman filter cards upon which they could prepare DBS. Canada has been a leader in advocating use of DBS for tracking COVID immunity. Protocols for collection, processing and optimal platforms for measurement were initiated early in the pandemic (22, 23) and several large surveys including those by Statistics Canada (24) and others (25, 26) were carried out with this flexible and user-friendly technique. DBS has significant advantages due to its low cost and wide range of potential analytes (27, 28). Our study provided a head-to-head comparison of these different procurement methods. Despite some modifications required for sample elution and measurement adjustment (12), both methods provided reliable measures and comparable results. This suggests that DBS may be an ideal method for monitoring of serologic responses in more remote geographic areas and other situations where venipuncture is not practical.

One other practical consideration underscored by this real-world study was the considerable between-batch heterogeneity in antibody measures. Sera and DBS were measured separately and temporally when the sampling was performed (**Figure 1**). We hypothesized that this heterogeneity was partly due to the design of our natural experiment, with participants who provided samples earlier in the study, and thus falling in earlier batches, were subject to Quebec’s initial policy of a longer dose interval. Similarly, participants in later batches correlated with those who received third doses, and therefore higher antibody levels. Batch-to-batch and lot-to-lot variability is well recognized in laboratory medicine and calibration steps are conducted to be ensure that measurements are comparable. Our models accounted for this variability, which is an important consideration when dealing with large scale quantitative studies. For example, a recent meta-analysis of antibody levels following vaccination was unable to determine a correlation of protection due to the large amount of heterogeneity between studies (6). Other factors may contribute to batch-to-batch variability, such as sample dilution in the era post vaccine, when dilution of 1000-10,000 fold may be required; this can amplify small variations in measurement. Thus, consideration of batch effects in our model was important for ensuring harmonization of all results despite measurement taking place over almost a 12-month period. This finding also highlights the need for studies to incorporate methods to decrease between-batch heterogeneity in the design phase of their study.

The immunologic basis for higher peak antibody levels with extended periods between doses is likely multifactorial. Following the initial dose of vaccine, antibody production generally peaks at 28 days. Reimmunizing at 21-28 days, as in the initial clinical trials (4, 5) risks having some of the antigen from the second vaccination dose neutralized by this pre-existing antibody, whereas re-vaccination when antibodies are waning diminishes this possibility (29). Secondly, the success of the second and subsequent doses likely depends on the presence of antigen in germinal centers. The interaction between B-cells and antigen presented by follicular dendritic cells continues long after the peak of antibody production (30). Re-exposure to the same antigen without sufficient time for previous antigen levels to diminish may also lead to suboptimal responses (30, 31). Responses to the third vaccine dose may be improved compared to the second of the primary series of vaccines due to higher baseline antibody levels. However, this may also be influenced by improved B-cell memory responses in those immunized with a longer interval (30) which is under investigation in our laboratory. The impact of longer intervals between vaccines on responses to the evolving variants of SARS-CoV2 also requires study. We have recently demonstrated using sera from our cohort that third and fourth doses of earlier COVID-19 vaccines (ancestral and/or BA4/BA5) can induce neutralizing antibodies to XBB1.5 (32). This may be a function of dose interval as well as multiple exposures to SARS-CoV2 spike proteins via repeat vaccinations.

The population we studied, health care workers and researchers, reflected a relatively healthy population. Our study does not directly provide evidence of the effectiveness of this approach in a pediatric population, in the frail elderly population or in immune compromised individuals; other cohorts have suggested similar results in the elderly (16). In addition to the immunologic outcomes that we followed, vaccine efficacy studies in Canada indicated that COVID outcomes such as hospitalization, severe diseases and mortality were comparable if not better with longer intervals between the primary two doses than similar jurisdictions which utilized standard vaccine protocols (33–35).

The recent CDC guidelines for future COVID-19 vaccination suggest a delay of 4-8 weeks between primary doses for mRNA vaccines depending on the subjects’ ages. Longer intervals may decrease risk of myocarditis and pericarditis associated with vaccination (36). Our data not only supports this recommendation, but potentially supports longer intervals between the primary doses as we continue to shield the populations against future waves of COVID-19 infection. In addition, our study has important implications for future vaccination campaigns in low and middle- income countries, where vaccine supply issues are an important public health challenge. To date, only 30% of people in low-income countries have received even a single dose of COVID vaccination (37). Clearly, our data indicate that delays outside of FDA approved protocols can be utilized for catch up vaccination of these important jurisdictions. Indeed, our methodologically strong study strengthens the body of evidence underscoring the value of increasing the interval between vaccine doses, which would allow a larger number of people to receive at least one dose of the vaccine. This strategy, used in Quebec and British Columbia (34), would help to maximize the immediate impact of available vaccines while buying additional time to secure more vaccine doses, and should be applied to future vaccine campaigns in both high and low resource settings.

### Limitations of the study

Our study cohort included health care workers and laboratory personnel and thus had a larger percentage of the females. The nature of the prospective-longitudinal study making the withdrawal rate and collection of samples unpredictable. We unfortunately did not have a 21- or 28-day vaccine control group readily available in Montreal, as only frail seniors in LTC were eligible for that. Despite extensive antibody studies we cannot predict any correlate of protection from this cohort.

## Conclusion

Our study confirms that mRNA COVID-19 vaccines are highly effective in inducing strong antibody responses in real-world conditions among health care personnel, first responders, and other essential workers. The strategy of a 16 week delay in the primary series was associated with higher antibody responses affecting all subsequent time points including the 3^rd^ vaccine/booster dose.

## Data availability statement

The raw data supporting the conclusions of this article will be made available by the authors, without undue reservation.

## Ethics statement

The studies involving humans were approved by The Research Ethics Board of the McGill University Health Centre (Protocol Numbers 2021-6747 and 2021-7534). The studies were conducted in accordance with the local legislation and institutional requirements. The participants provided their written informed consent to participate in this study.

## Author contributions

NA: Conceptualization, Formal analysis, Investigation, Writing – original draft. IS: Formal analysis, Writing – review & editing, Data curation, Methodology, Validation. DK: Formal analysis, Project administration, Writing – review & editing. ES: Writing – original draft. MPl: Data curation, Investigation, Project administration, Supervision, Writing – review & editing. SV: Data curation, Project administration, Supervision, Writing – review & editing. AM: Investigation, Project administration, Writing – review & editing. MB: Data curation, Investigation, Project administration, Writing – review & editing. CL: Investigation, Writing – review & editing. NW: Data curation, Investigation, Writing – review & editing. JF: Conceptualization, Investigation, Validation, Writing – review & editing. CP: Conceptualization, Validation, Writing – review & editing. MPe: Formal analysis, Software, Writing – review & editing. CA: Formal analysis, Investigation, Methodology, Writing – review & editing. YG: Investigation, Methodology, Writing – review & editing. PM: Investigation, Methodology, Writing – review & editing. ML: Conceptualization, Formal analysis, Investigation, Methodology, Validation, Writing – original draft, Writing – review & editing. KD: Conceptualization, Formal analysis, Methodology, Writing – original draft, Writing – review & editing. BM: Conceptualization, Formal analysis, Funding acquisition, Project administration, Supervision, Writing – original draft, Writing – review & editing.

## Supplementary material

Supplementary Figure 1 Between-dose interval distribution of the study population. Distribution of the delay in days between the first and second doses on the primary COVID-19 vaccines.

Supplementary Figure 2 Association of batch with between-dose interval (long *vs* short), time since vaccine (visit), and IgG anti-RBD (BAU/ml), serum cohort. This figure shows IgG-RBD values (BAU/ml) for each of the 7 batches, stratified by long and short dose interval. Time since vaccine (visit) is indicated by the shape of the data points. Within each batch, participants in the short interval group (red) lagged behind the long interval group (blue) in terms of visit. This is especially striking for batch B05, where the short interval group is mainly composed of participants at 3 months post second dose (triangles) while the long interval has a majority of participants at 6 months post 2nd dose (squares). This may be an artifact of Quebec’s vaccine policy where those who were the first to receive the vaccine (and therefore fell in earlier batches) were subject to longer dose intervals. The fact that, within each batch, the short interval group included participants who had received their last vaccine dose more recently than those in the long interval group explains why the short interval group has higher mean antibody levels *vs* the long interval group for each batch. This between-batch heterogeneity in antibody levels, time since vaccine and between-dose intervals made it imperative to control for batch in our multivariate models.

Supplementary Figure 3 Antibody data from Dried Blood Spot (DBS) samples: IgG antibodies levels of anti-Spike **(A)**, anti-RBD **(B)**, anti-N **(C)**, at 28 days post-2^nd^ dose, 3- and 6 months post-2^nd^ dose and 28 days post-3^rd^ vaccine dose, DBS cohort. Univariate analysis, the values are BAU/mm^2^.
