# Supplementary figures and images for: The effect of dose-interval on antibody response to mRNA COVID-19 vaccines: a prospective cohort study
Source: Front Immunol. 2024 Feb 16;15:1330549. doi: 10.3389/fimmu.2024.1330549 (PMC10904688; doi:10.3389/fimmu.2024.1330549)

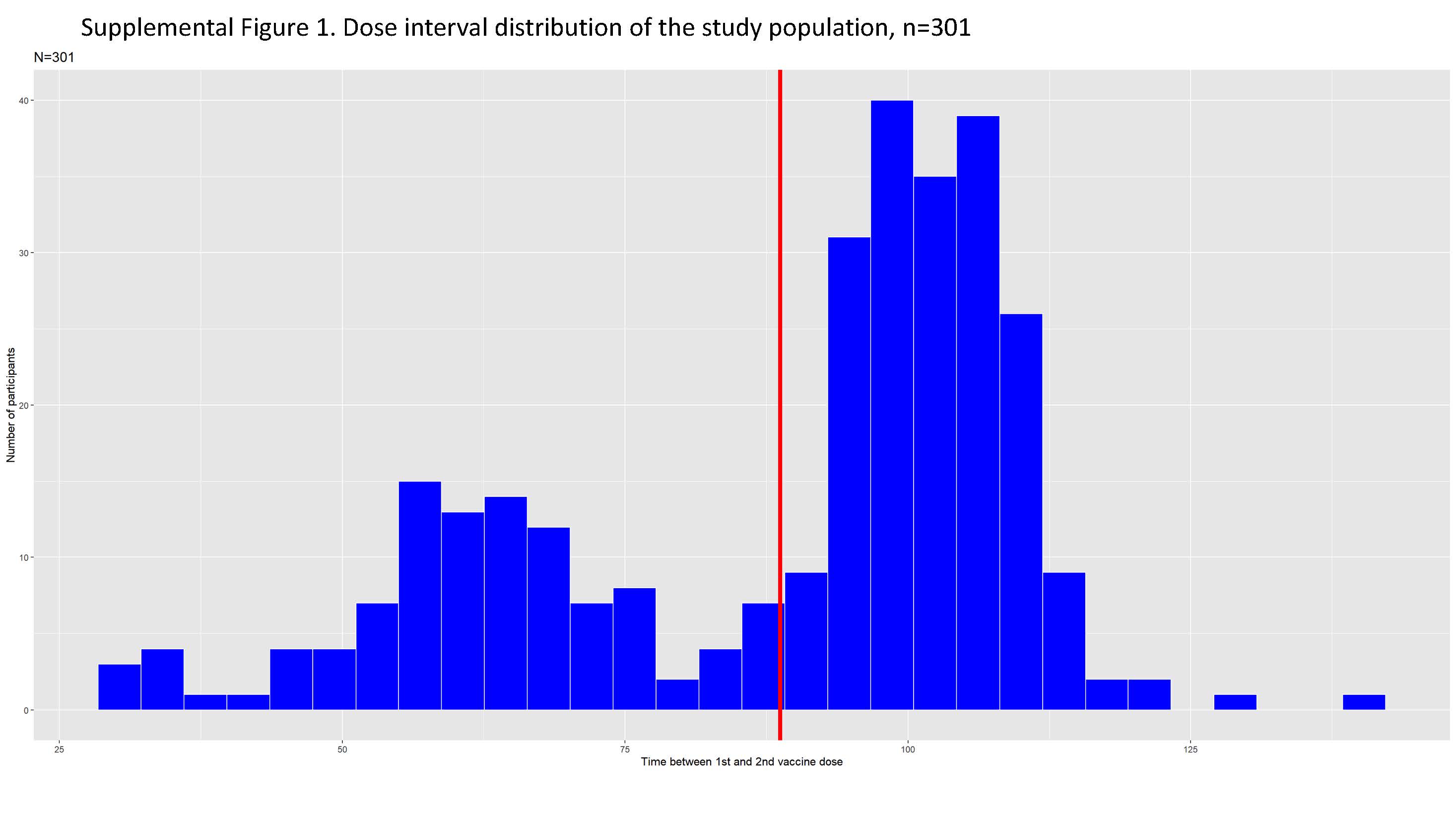

Supplement: Supplementary Figure 1 — Between-dose interval distribution of the study population. Distribution of the delay in days between the first and second doses on the primary COVID-19 vaccines. [file Image_1.jpeg]

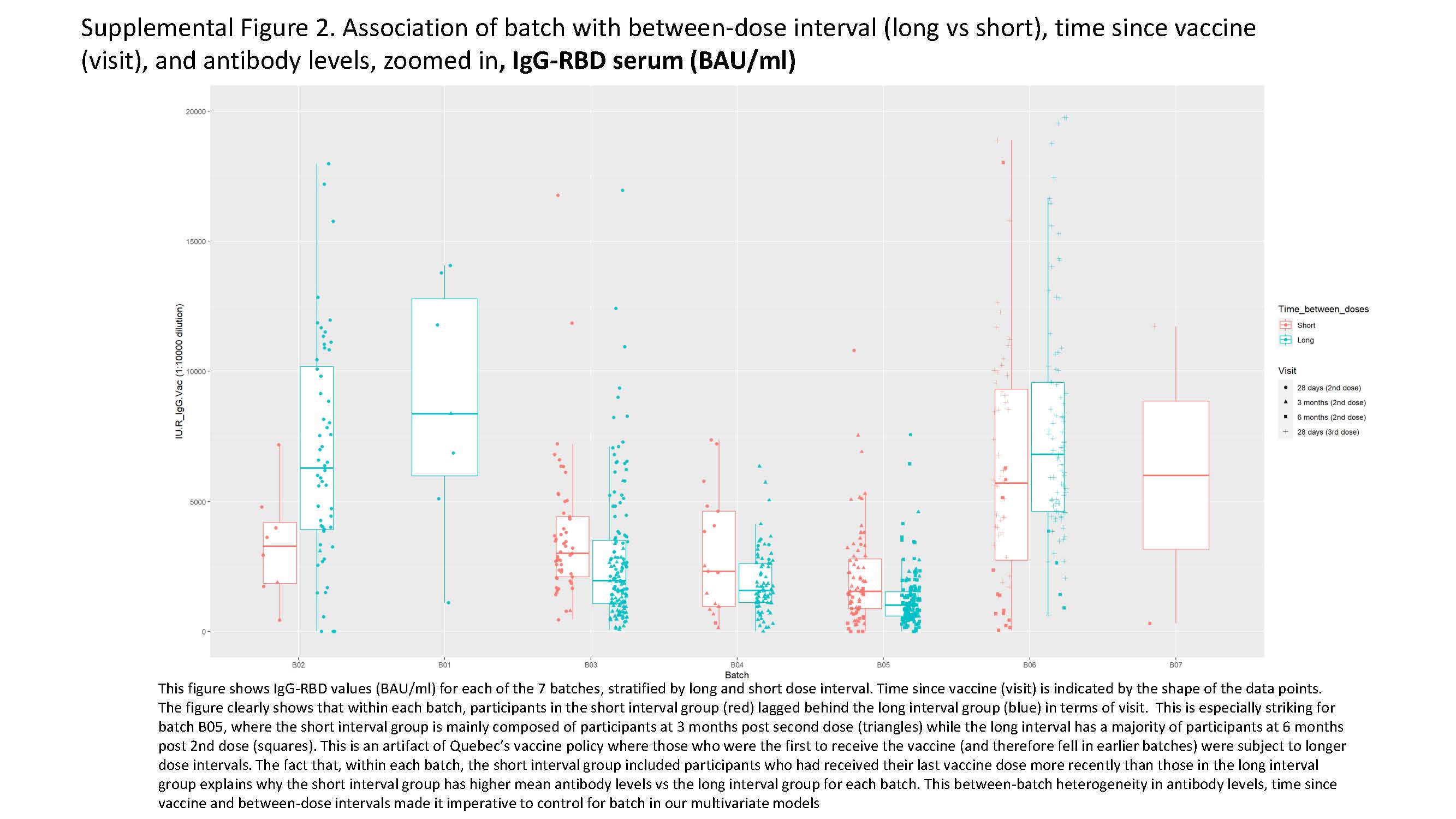

Supplement: Supplementary Figure 2 — Association of batch with between-dose interval (long vs short), time since vaccine (visit), and IgG anti-RBD (BAU/ml), serum cohort. This figure shows IgG-RBD values (BAU/ml) for each of the 7 batches, stratified by long and short dose interval. Time since vaccine (visit) is indicated by the shape of the data points. Within each batch, participants in the short interval group (red) lagged behind the long interval group (blue) in terms of visit. This is especially striking for batch B05, where the short interval group is mainly composed of participants at 3 months post second dose (triangles) while the long interval has a majority of participants at 6 months post 2nd dose (squares). This may be an artifact of Quebec’s vaccine policy where those who were the first to receive the vaccine (and therefore fell in earlier batches) were subject to longer dose intervals. The fact that, within each batch, the short interval group included participants who had received their last vaccine dose more recently than those in the long interval group explains why the short interval group has higher mean antibody levels vs the long interval group for each batch. This between-batch heterogeneity in antibody levels, time since vaccine and between-dose intervals made it imperative to control for batch in our multivariate models. [file Image_2.jpeg]

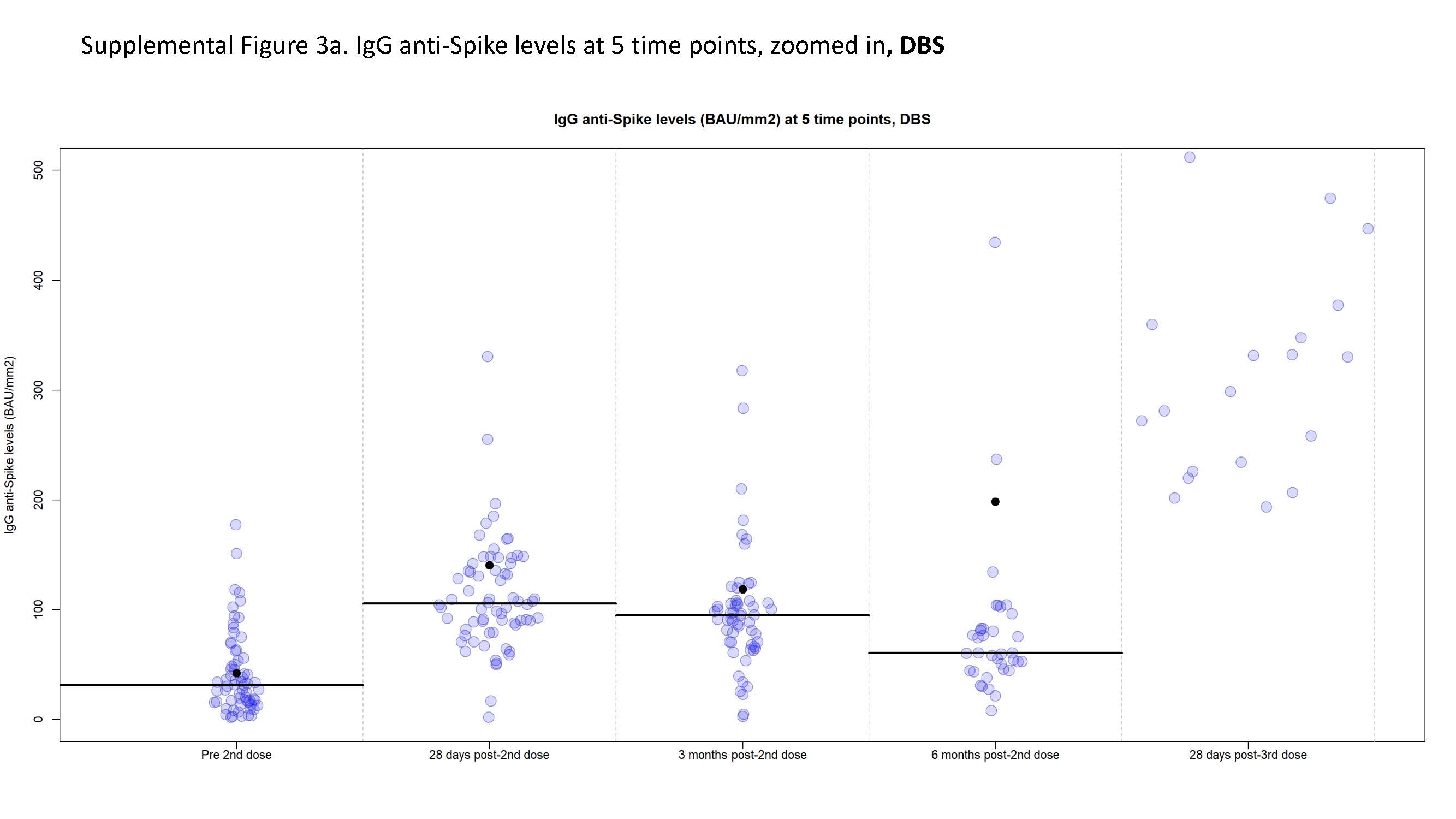

Supplement: Supplementary Figure 3 — Antibody data from Dried Blood Spot (DBS) samples: IgG antibodies levels of anti-Spike (A), anti-RBD (B), anti-N (C), at 28 days post-2nd dose, 3- and 6 months post-2nd dose and 28 days post-3rd vaccine dose, DBS cohort. Univariate analysis, the values are BAU/mm2. [file Image_3.jpeg]

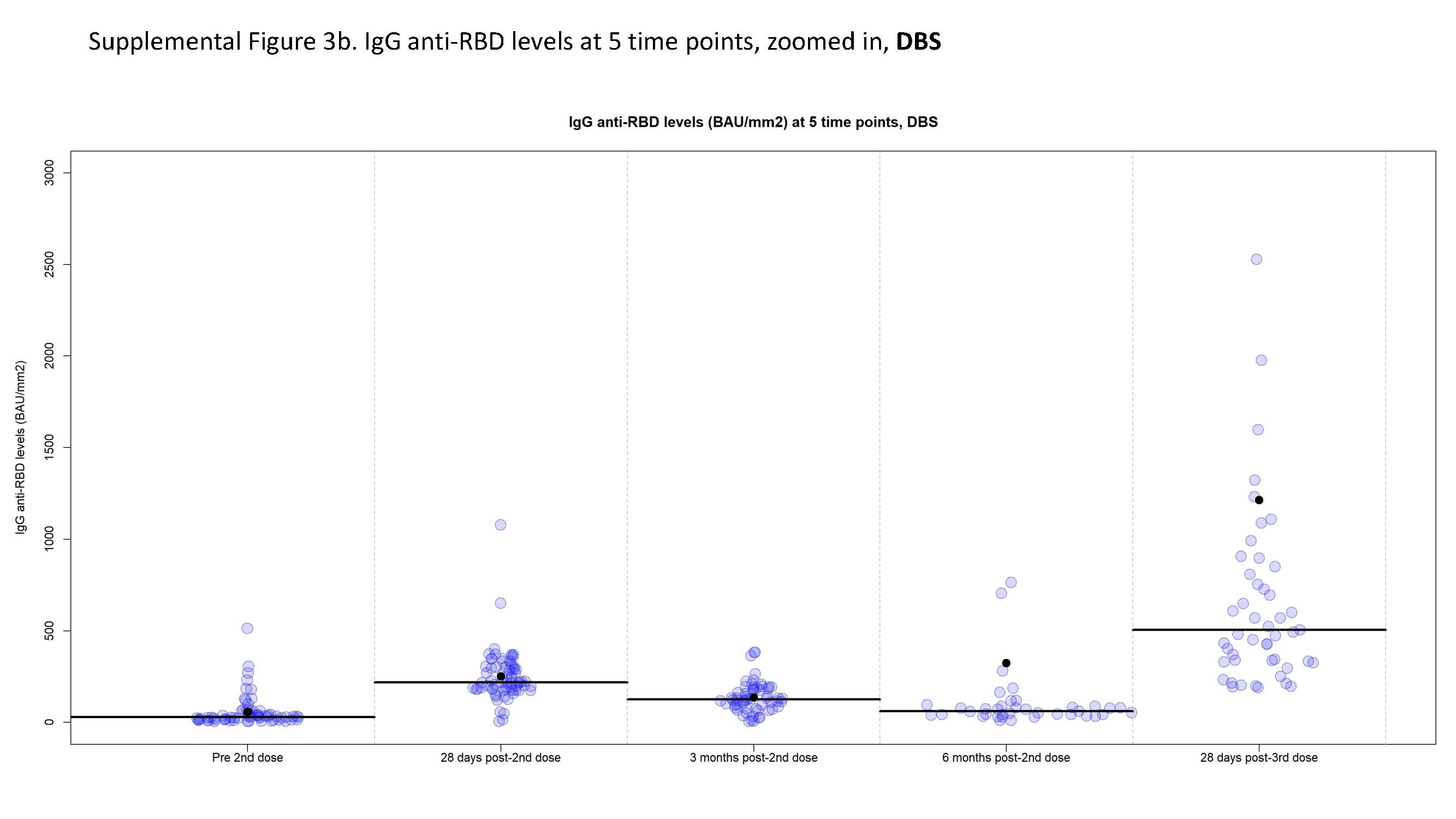

Supplement: Supplementary file 4 [file Image_4.jpeg]

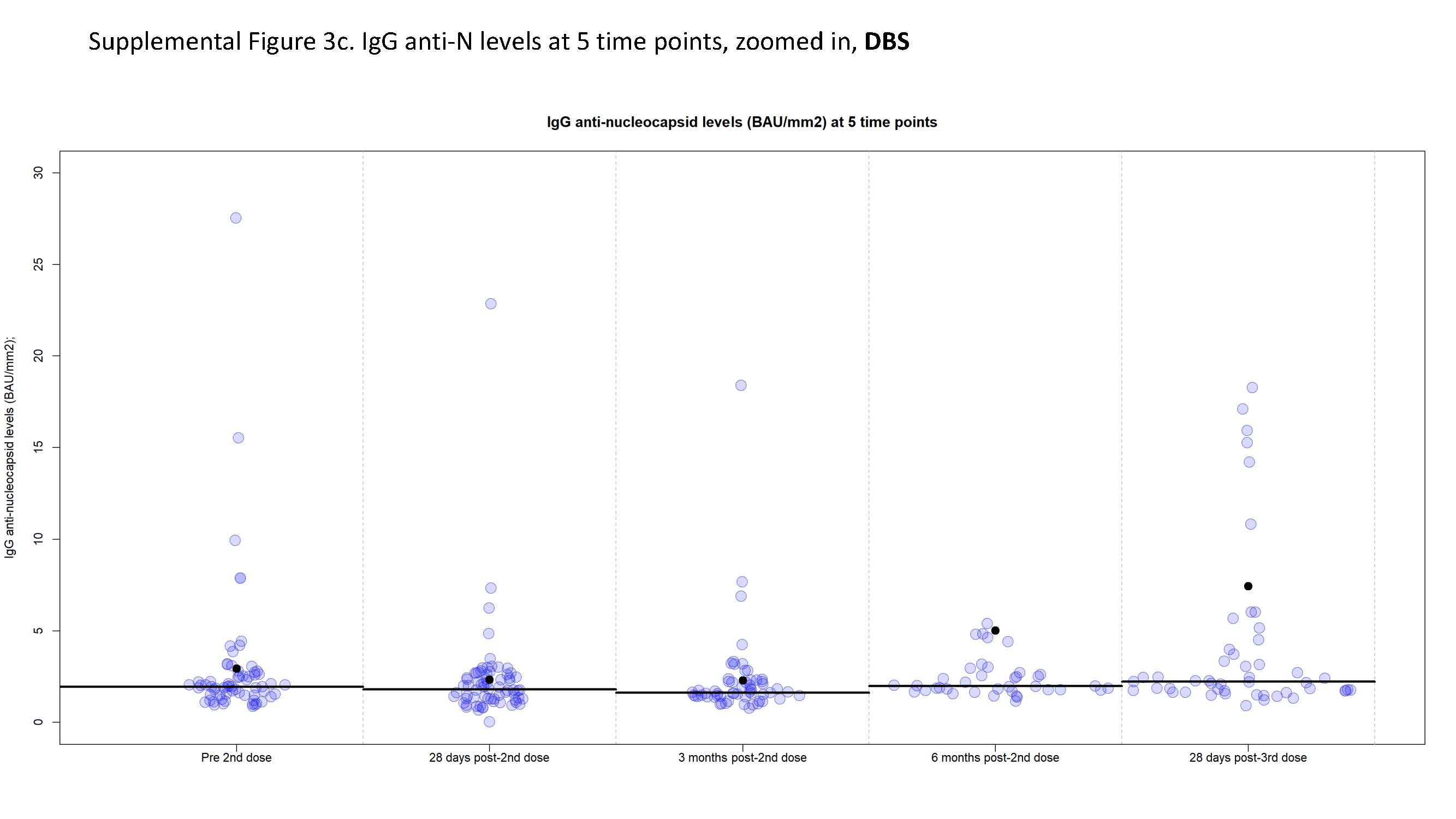

Supplement: Supplementary file 5 [file Image_5.jpeg]
