# Supplementary material for: The effect of dose-interval on antibody response to mRNA COVID-19 vaccines: a prospective cohort study
Source: Front Immunol. 2024 Feb 16;15:1330549. doi: 10.3389/fimmu.2024.1330549 (PMC10904688; doi:10.3389/fimmu.2024.1330549)
Supplement: Supplementary file 6 [file DataSheet_1.pdf]

Supplemental Table 1. Median, arithmetic and geometric mean antibody levels at 28 days post-2nd dose, 3 months post-2nd dose, 6 months post-2nd dose and 28-days post-3rd dose, Serum and DBS cohorts

	28-day post 2nd dose visit			3 months post 2nd dose			6 months post 2nd dose			28-day post 3rd dose visit		
	Median (IQR)	Mean (SD)	Geom. Mean (SD)	Median (IQR)	Mean (SD)	Geom. Mean (SD)	Median (IQR)	Mean (SD)	Geom. Mean (SD)	Median (IQR)	Mean (SD)	Geom. Mean (SD)
**SERUM**												
**IgG RBD**,	3898.0	6168.6	3992.4	1564.7	1830.7	1366.4 (2.4)	929.0	1256.7	792.3	7353.4	11722.4	7782.6
BAU/ml	(4374.6)	(9833.0)	(2.5)	(1411.3)	(1371.2)		(888.8)	(1666.9)	(2.9)	(5989.5)	(18057.3)	(2.2)
**IgG Spike**,	3572.6	6085.4	3799.5	1564.7	1897.4	1498.5	1139.4	1487.3	1089.5	7934.5	11256.7	8604.9
BAU/ml	(4724.4)	(7364.4)	(2.6)	(1411.3)	(1432.2)	(2.1)	(834.6)	(1858.4)	(2.3)	(9268.9)	(8591.4)	(2.1)
**IgG N,**	2.3	5.8	1.6	2.8	5.8	1.7	3.4	7.3	2.5	6.0	19.7	6.2
BAU/ml	(6.4)	(10.6)	(6.8)	(6.5)	(11.1)	(6.2)	(7.4)	(12.1)	(5.7)	(15.9)	(34.0)	(5.3)
**Neutralizing**	1716.7	1737.2	1077.9	274.7	552.2	216.4	53.2	232.3	42.7	3777.9	3336.0	2937.5
Ab, IU/ml	(1966.9)	(1127.5)	(4.1)	(605.1)	(698.1)	(5.5)	(120.5)	(637.4)	(6.1)	(940.6)	(1077.5)	(2.0)
**DBS**												
**IgG RBD**,	217.6	250.8	210.7	125.4	135.5	104.5	61.5	323.7	73.3	505.0	1213.7	629.1
BAU/mm^2	(122.2)	(143.6)	(2.1)	(102.0)	(82.8)	(2.4)	(44.9)	(1373.9)	(3.2)	(566.0)	(2086.9)	(2.7)
**IgG Spike**,	105.6	140.5	103.6	95.0	118.5	84.5	60.6	198.3	80.1	652.3	1021.8	747.5
BAU/mm^2	(54.6)	(233.9)	(2.1)	(37.1)	(165.9)	(2.3)	(51.4)	(448.7)	(2.9)	(1657.3)	(738.4)	(2.3)
**IgG N,**	1.8	2.3	1.8	1.6	2.3	1.8	2.0	5.0	2.5	2.2	7.4	3.2
BAU/mm^2	(1.3)	(2.8)	(2.2)	(0.8)	(2.5)	(1.7)	(0.9)	(16.3)	(2.1)	(2.5)	(17.8)	(2.7)

Supplemental Table 2. Multivariable regression analyses demonstrating the association of between-dose interval with antibody levels for participants with serum and DBS samples, separately (estimates as percent change after back-transforming betas)

	Serum (n=743)			DBS (n=216)	
Variables in the regression model	**IgG RBD** β (95% CI)*	**IgG Spike** β (95% CI)*	**Neutralizing Ab** β (95% CI)*	**IgG RBD** β (95% CI)*	**IgG Spike** β (95% CI)*
**Interval**					
Long	27.7 (3.2, 58.0)	24.0 (4.1, 47.6)	34.9 (-2.7, 87.0)	66.2 (16.8, 136.5)	64.6 (16.4, 132.6)
Short	Reference	Reference	Reference	Reference	Reference
**Age, 1-year increments**	-1.7 (-2.4, -1.0)	-1.1 (-1.6, -0.5)	-2.6 (-3.6, -1.6)	-0.6 (-1.8, 0.7)	-0.3 (-1.6, 1.0)
**Sex**					
Female	36.5 (11.1, 67.7)	25.3 (5.9, 48.3)	51.5 (10.5, 107.5)	-3.8 (-32.0, 36.2)	4.7 (-26.1, 48.5)
Male	Reference	Reference	Reference	Reference	Reference
**Infection status**					
Prior infection	66.4 (34.3, 106.2)	53.2 (28.6, 82.4)	87.1 (35.4, 158.7)	68.4 (23.0, 130.4)	73.6 (28.0, 135.2)
Naive	Reference	Reference	Reference	Reference	Reference
**Time since vaccine dose**					
3 months post 2nd dose	-66.6 (-72.2, -59.9)	-62.5 (-67.9, -56.3)	-81.8 (-86.4, -75.8)	-54.8 (-70.5, -31.5)	-38.3 (-57.7, -11.3)
6 months post 2nd dose	-86.8 (-90.4, -81.8)	-83.2 (-87.1, -78.0)	-97.2 (-98.3, -95.5)	-75.5 (-86.9, -54.4)	-64.5 (-78.9, -38.6)
28 days post 3rd dose	-17.0 (-50.4, 38.7)	-7.9 (-40.2, 41.7)	-71.6 (-87.3, -36.8)	113.7 (18.9, 282.0)	233.0 (98.2, 454.2)
28 days post 2nd dose	Reference	Reference	Reference	Reference	Reference
**Batch**					
1	139.3 (43.2, 300.0)	114.4 (38.9, 230.8)	0.3 (-55.1, 123.7)	-1.2 (-36.3, 52.3)	-7.1 (-36.8, 35.1)
3	-19.7 (-35.9, 0.2)	-52.8 (-60.9, -43.1)	172.1 (92.0, 284.9)	27.0 (-19.5, 95.0)	81.8 (26.1, 165.9)
4	-16.5 (-37.0, 10.7)	-36.7 (-50.1, -19.7)	62.6 (4.7, 152.8)	-	-
5	35.5 (-4.6, 92.6)	5.0 (-21.9, 41.2)	122.4 (28.6, 284.9)	-	-
6	92.9 (10.9, 235.8)	45.7 (-8.5, 132.2)	1598.9 (617.0, 3936.8)	-	-
7	475.6 (151.0, 1221.8)	157.9 (28.2, 419.1)	940.3 (185.0, 3710.2)	-	-
2	Reference	Reference	Reference	Reference	Reference

* Separate regression models evaluating the impact of dose interval (long vs. short) on log antibody response were run for each of the three antibodies, expressed as BAU/ml for serum and BAU/mm^2 for DBS. Models were adjusted for age as a continuous variable, sex, prior infection status, batch of analysis, and timepoint. Estimates (and 95% confidence intervals shown in parentheses) represent the percent change in back-transformed antibody levels [$(2^{\beta}$ -1)*100] comparing long vs. short interval groups.

Supplemental Table 3. Multivariable regression analyses demonstrating the association of between-dose interval with log antibody levels for participants with serum and DBS samples, separately (original beta estimates)

	Serum (n=743)			DBS (n=216)	
Variables in the regression model	**IgG RBD** β (95% CI)*	**IgG Spike** β (95% CI)*	**Neutralizing Ab** β (95% CI)*	**IgG RBD** β (95% CI)*	**IgG Spike** β (95% CI)*
**Interval**					
Long	0.353 (0.046, 0.660)	0.310 (0.058, 0.562)	0.432 (-0.039, 0.903)	0.733 (0.224, 1.242)	0.719 (0.219, 1.218)
Short	Reference	Reference	Reference	Reference	Reference
**Age, 1-year increments**	-0.025 (-0.035, -0.015)	-0.016 (-0.024, -0.007)	-0.038 (-0.054, -0.023)	-0.008 (-0.027, 0.010)	-0.004 (-0.023, 0.014)
**Sex**					
Female	0.449 (0.152, 0.746)	0.326 (0.083, 0.568)	0.599 (0.144, 1.053)	-0.056 (-0.557, 0.446)	0.066 (-0.437, 0.571)
Male	Reference	Reference	Reference	Reference	Reference
**Infection status**					
Prior infection	0.752 (0.447, 1.058)	0.615 (0.363, 0.867)	0.904 (0.437, 1.371)	0.752 (0.299, 1.204)	0.796 (0.356, 1.234)
Naive	Reference	Reference	Reference	Reference	Reference
**Time since vaccine dose**					
3 months post 2nd dose	-1.577 (-1.843, -1.312)	-1.417 (-1.641, -1.193)	-2.461 (-2.877, -2.047)	-1.146 (-1.761, -0.545)	-0.697 (-1.240, -0.173)
6 months post 2nd dose	-2.915 (-3.375, -2.456)	-2.569 (-2.957, -2.183)	-5.182 (-5.902, -4.466)	-2.030 (-2.938, -1.134)	-1.493 (-2.302, -0.704)
28 days post 3rd dose	-0.255 (-0.995, 0.484)	-0.119 (-0.742, 0.503)	-1.815 (-2.973, -0.663)	1.096 (0.249, 1.934)	1.735 (0.987, 2.470)
28 days post 2nd dose	Reference	Reference	Reference	Reference	Reference
**Batch**					
1	1.259 (0.518, 2.000)	1.100 (0.474, 1.726)	0.004 (-1.156, 1.162)	-0.017 (-0.650, 0.607)	-0.106 (-0.661, 0.435)
3	-0.317 (-0.639, 0.003)	-1.084 (-1.356, -0.814)	1.444 (0.941, 1.945)	0.345 (-0.261, 0.964)	0.862 (0.334, 1.411)
4	-0.261 (-0.668, 0.146)	-0.660 (-1.003, -0.316)	0.701 (0.066, 1.338)	-	-
5	0.438 (-0.069, 0.945)	0.071 (-0.356, 0.498)	1.153 (0.362, 1.942)	-	-
6	0.948 (0.150, 1.747)	0.543 (-0.128, 1.216)	4.087 (2.842, 5.335)	-	-
7	2.525 (1.328, 3.724)	1.367 (0.358, 2.376)	3.379 (1.511, 5.252)	-	-
2	Reference	Reference	Reference	Reference	Reference

* Separate regression models evaluating the impact of dose interval (long vs. short) on log antibody response were run for each of the three antibodies, expressed as BAU/ml for serum and BAU/mm^2 for DBS. Models were adjusted for age as a continuous variable, sex, prior infection status, batch of analysis, and timepoint. Estimates (and 95% confidence intervals shown in parentheses) represent the percent change in back-transformed antibody levels [$(2^{\beta}$ -1)*100] comparing long vs. short interval groups.
